# Supplementary material for: Self-replication of DNA by its encoded proteins in liposome-based synthetic cells
Source: Nat Commun. 2018 Apr 20;9:1583. doi: 10.1038/s41467-018-03926-1 (PMC5910420; doi:10.1038/s41467-018-03926-1)
Supplement: Supplementary file 1 — Supplementary Information [file 41467_2018_3926_MOESM1_ESM.pdf]

Supplementary Information for

Self-replication of DNA by its encoded proteins in liposome-based synthetic cells

van Nies et al.

SUPPLEMENTARY METHODS

***In vitro* transcription and RNA purification**

Transcription of DNA templates was performed with the RiboMAX^{TM} Large Scale RNA production kit (Promega) according to the protocol recommended by the supplier. Alternatively, the PURE*frex* devoid of ribosome (ΔR) was used. RNA purification was carried out using the RNeasy MinElute Cleanup kit (Qiagen) following the manufacturer's protocol. The concentration of RNA was determined by absorbance measurements at 280 nm using a Nanodrop (Thermo Scientific). After purification, the RNA samples were imaged on a 1.2% agarose gel containing EtBr and the band intensities were analyzed with ImageLab.

Rolling circle amplification assays

The M13mp18ss (New England Biolabs) circular ssDNA plasmid was used as template for the RCA reactions in combination with the hybridization primer 5'-GTTTTCCCAGTC ACGAC-3' (with or without 5'-Cy5 label). To pre-hybridize the primer to the template, the M13mp18ss and primer were mixed in nuclease-free water in molar ratio 1:5, preheated for 5 min at 65 °C and cooled down at room temperature for 5 min. The final 25-µL solution contains 20 units Φ29 DNAP (New England Biolabs), 1× Φ29 DNA Polymerase Reaction Buffer (50 mM Tris-HCl, 10 mM $MgCl_2$, 10 mM ammonium sulfate, 4 mM DTT, pH 7.5 at 25 °C), 0.3 mM dNTP mix, 0.02 µM template and 0.1 µM primer. Samples with NTPs were supplemented with 3 mM ATP, 3 mM GTP, 1 mM CTP and 1 mM UTP. When the activity of the PURE*frex*-expressed DNAP was tested, the above protocol was employed except that the phi29 buffer was omitted and the purified Φ29 DNAP was replaced by 3 nM of the *p2* DNA template added to the PURE*frex* reaction solution. Volumes of 5 µL were harvested at the indicated time-points and the reaction was quenched with 2.5 µL of the STOP solution (final concentrations: 10 mM EDTA, 0.1% SDS) supplemented with 0.5 µL of 0.1 mg/mL Proteinase K solution. The samples were loaded on a 0.7% alkaline agarose gel and after-stained with EtBr.

DNAP activity assay using a linear ssDNA precursor of coding dsDNA

The ssDNA *eYFPco-LL-Spinach* template was produced in two steps. First, a PCR reaction was performed on the plasmid containing the *T7p-eYFPco-LL-Spinach-T7t* gene, using the forward primer ChD185 and reverse primer ChD190 (Supplementary Table 2). The resulting *eYFPco-LL-Spinach* dsDNA construct lacks a T7 promoter and contains a poly-A tail instead of the T7 terminator. The purified DNA product was subsequently used as input for an asymmetric PCR reaction, where the forward primer was diluted 50 times with respect to reverse primer to generate the ssDNA *ΔT7p-eYFPco-LL-Spinach-polyA* construct asides the corresponding dsDNA.

The *ΔT7p-eYFPco-LL-Spinach-polyA* ssDNA was hybridized to primer ChD361 (Supplementary Table 2) that contains the T7 promoter sequence and an overhang for hybridization. Hybridization was performed for 5 min at 65 °C, prior to supplementing the DNA to the PURE system reaction. A standard PURE*frex* reaction was supplemented with 200 µM dNTP mixture (Promega), 0.5 µL of 20 U/µL SUPERase inhibitor (10 U final; Ambion), 20 µM DFHBI (final concentration), 200 ng ss/dsDNA of *ΔT7p-eYFPco-LL-*

Spinach-polyA, 1 µM of primer ChD361 (final concentration) and either 3.6 nM of *p2* DNA, 5 units of purified Φ29 DNAP (New England Biolabs), or milliQ water in the control reaction. The reaction mixtures were transferred to 15-µL cuvettes (Hellma) that were mounted in the temperature-controlled holder of a fluorescence spectrophotometer (Cary Eclipse from Varian) held at 37 °C and the fluorescence was recorded every 30 s using the following excitation/emission wavelengths: Spinach, 460/502 nm; YFP, 515/528 nm.

Amplification of the Φ29 genome with purified or/and PURE*frex*-synthesized proteins
In the experiments where amplification of the Φ29 genome was also mediated by purified proteins (Supplementary Fig. 7), the starting amount of Φ29 genome was 95 ng (0.4 nM) and the amounts of purified proteins that were supplemented to the standard 20-µL PURE*frex* reaction were: 40 ng p2, 40 ng p3, 2 µg p6 and 7.5 µg p5. Alternatively, the p2 and p3 proteins, and the p5 and p6 proteins were synthesized from 0.4 nM *oriLR-p2-p3* template and from 1.5 nM *oriLR-p5-p6* template, respectively.

Elongation of primed ssDNA by synthesized Φ29 DNAP in liposome-confined reactions
Lipid-coated beads were prepared as follows. First, a mixture containing 1 mg DOPC, 0.25 mg DOPG, 0.06 mg DSPE-PEG(2000)-biotin (all from Avanti Polar Lipids) and 0.0125 mg DHPE-Texas Red (Invitrogen), dissolved in chloroform, was prepared. The corresponding lipid fraction is DOPC 79 mol. %, DOPG 19.75 mol. %, DSPE-PEG-biotin 1.25 mol. % and DHPE-Texas Red 0.95 mass %. This mixture was then deposited drop-by-drop on 1 g of glass beads (212 µm-300 µm, acid washed, Sigma-Aldrich), preheated to ~60 °C. After lipid deposition, residual chloroform was removed by 2 h of desiccation. Lipid-coated beads were stored under argon to protect the lipids from oxidative damage and can be used for at least one month without compromising liposome quality.

For the liposome experiments, a 19-µL standard PURE*frex*2.0 reaction was supplemented with 200 µM dNTP mixture (Promega), 0.5 µL of 20 U/µL SUPERase inhibitor, 100 nM PTH2 (Hölzel Diagnostika Handels GmbH, Germany), 222 ng *ΔT7p-eYFPco-LL-Spinach-polyA* DNA hybridized with 1 µM primer ChD361 as described above. The solution was split in two 9.5-µL aliquots, one supplemented with 1.3 nM *p2* DNA, the other with an equal volume of milliQ (negative control). To both 10-µL reactions, 7.5 mg of lipid-coated beads was added. Liposomes were formed by spontaneous swelling of the lipid film for 2 h on ice, during which both samples are subjected to regular tumbling.

Custom-made glass reaction chambers were coated first with a mixture of BSA and BSA-biotin, and then with Neutravidin to provide an immobilization surface for the biotinylated liposomes. About 2 µL of the liposome solution was pipetted (with a cut tip to avoid damaging the liposomes) in the reaction chamber. After ~60 s of immobilization, 5 µL of feeding solution, consisting of a 1:1 vol of PURE*frex*2.0 Solution I and buffer (50 mM HEPES, 100 mM potassium glutamate, pH 7.6), supplemented with 33 µM DFHBI and 0.25 mg/mL proteinase K, was added to the liposome solution in the reaction chamber. The reaction was incubated overnight at 37 °C, after which imaging of the samples was performed with a Nikon A1R laser scanning confocal microscope. During image acquisition, the sample temperature was maintained at 37 °C. Acquisition was performed with the following

excitation/emission wavelengths: 457/482 nm (Spinach), 514/540 nm (YFP), and 561/595 nm (Texas Red).

SUPPLEMENTARY NOTES

Supplementary Note 1: *De novo* design of linear DNA constructs coding for Φ29 replication proteins

The design strategy satisfies the following requirements: (i) The linear DNA to be amplified is flanked by two sequences that are the Φ29 replication origins, either the minimal 68-bp origins, or the 191-bp left and 194-bp right end origins. (ii) The constructs contain a terminal 5'-phosphate group. (iii) Transcription of all individual genes, including in the dual-gene constructs (two genes being assembled as two-cistron transcriptional units) is under control of the T7 promoter followed by the g10-leader sequence of the T7 genome. (iv) To terminate transcription, the natural T7 terminator sequence was introduced at the end of the construct. In the case of the dual-gene templates, a tandem repeat of the vesicular stomatitis virus (VSV) terminator separated by a spacer of 16 bp was implemented in between the two genes. Termination efficiency in PURE system reactions was estimated to be 70% for the canonical T7 promoter and 80% for the VSV-tandem (Supplementary Fig. 2). A schematic of the two-cistron construct design is shown in Supplementary Fig. 1. All DNA constructs with their regulatory elements are reported in Supplementary Table 1. (v) Gene sequences were optimized for expression in the *E. coli*-based PURE system, which includes optimization of codon usage, GC content and mRNA secondary structure. All DNAs were chemically synthesized.

Supplementary Note 2: Sample analysis on alkaline and neutral agarose gels

Replication samples were regularly analysed on both an alkaline and on a neutral gel. The advantage of analysis on alkaline gels is that RNA present in the PURE system as rRNA, mRNA and tRNA, does not have to be removed by RNase treatment and column purification because it will undergo partial cleavage in the alkaline gel conditions. It will therefore not interfere with the DNA bands of interest. Moreover, the number of steps in the protocol is reduced making this analysis more robust. However, the disadvantage is that the gel shows only ssDNA species and it is therefore not possible to distinguish between the scenario of merely protein-primed strand displacement reaction events by the DNAP, or complete dsDNA amplification requiring initiation at both ends of the same DNA template. The advantage of analysis on a neutral gel is that an observed increase of the dsDNA band intensity is a direct evidence for DNA replication. The experimental difficulty lies in the need for the complete removal of all RNA species prior to imaging on gel. For this reason, it is also possible to visualize any by-products the reaction may produce, that would otherwise be blurred by small RNA molecules in the gel. The corresponding protocols are described in details in the Methods section of the main paper.

Supplementary Note 3: Validation of the activity of the synthesized Φ29 DNA polymerase

First, the strand-displacement activity and the high processivity of the synthesized DNAP were confirmed by conducting a rolling circle amplification assay (Supplementary Fig. 4). Second, the polymerase can elongate a primer-template junction to generate a transcriptionally active linear dsDNA and produce functional encoded proteins (Supplementary Fig. 5). Third, the ability of the PURE*frex*-synthesized TP and DNAP to form a DNA replication initiation complex was tested using the *oriL68-p2-p3* dsDNA template. The *de novo* synthesized DNA was visualized through the incorporation of fluorescent ribonucleotides, demonstrating successful protein-primed DNA production (Supplementary Fig. 6). We then sought to demonstrate that the Φ29 DNAP could be synthesized in an active state inside liposome. The gene for the Φ29 DNAP was co-encapsulated with an oligo-primed ssDNA that can merely serve as a template for IVTT and produce the YFP reporter if the synthesized Φ29 polymerase elongates the ssDNA into a transcriptionally active dsDNA template. A significant number of liposomes exhibit YFP signal demonstrating that the vesicle-confined transcription-translation reaction produces active Φ29 DNAP (Supplementary Fig. 17).

Supplementary Note 4: Estimation of the concentration of synthesized proteins

Co-translational insertion of fluorescently-labelled lysine can be used to visualize synthesized proteins by PAGE, even when their concentrations are lower than the detection limit with CBB staining (Fig. 1E). However, GreenLys is not a reliable method for protein quantitation because the insertion efficiency of a fluorescent lysine (in competition with unlabelled lysine) at a given position is largely unknown and it varies in the course of gene expression. Moreover, the band intensity depends on the occurrence of Lys residues in the primary sequence, which can differ from one protein to another. Therefore, we chose CBB staining in combination with purified protein standards to estimate the concentrations of synthesized proteins (Supplementary Fig. 3).

Supplementary Note 5: Estimation of the theoretical maximal amplification yield

The maximal fold-amplification of DNA substrate (in this study the Φ29 genome and the *oriLR-p2-p3* construct) can be estimated from the concentration of dNTPs, the nucleotide sequence and input amount of DNA. Starting from 140 ng of Φ29 genome (equiv. to 0.59 nM; Fig. 2) or from 140 ng of *oriLR-p2-p3* (equiv. to 3.5 nM; Supplementary Fig. 9) results in 44- and 47-times maximal predicted amplification, respectively.

Supplementary Note 6: Acridine orange as a DNA dye for amplification assays inside liposomes

Acridine orange (AO) was chosen as a DNA intercalating dye for three reasons. First, it has a low molecular weight compared to other standard DNA-binding fluorophores. Second, it is known as a membrane permeable dye, hence crossing the liposome membrane is highly possible. Third, its spectral properties are different when bound to DNA or RNA, an asset given the background of tRNA, rRNA and mRNA in PURE*frex*.

We noticed that AO could also stain the liposome membrane (Fig. 5B). We checked possible cross-talk between the membrane dye Texas-red and AO channels and could definitely rule out the possibility that Texas-red signal would leak into the AO channel. Due to the hydrophobic nature of AO it is likely that it also partitions in the lipid bilayer. Alternatively, RNAs in PURE*frex* (mRNA, tRNAs) stained with AO could interact with the liposome membrane. However, we expect that AO bound to RNA exhibits a larger fluorescence red-shift (Exc./Em. 460 nm/650 nm for RNA vs. 500 nm/526 nm for DNA) that would be incompatible with the settings of the selected green channel.

Supplementary Note 7: Alternative approaches to the Φ29 virus-based protein-primed DNA replication.

Unlike other mechanisms proposed earlier, the genomic DNA is not circular but linear, circumventing for instance the problem of DNA recombination to circularize the long linear concatemer product of RCA-based DNA synthesis [1,2]. In other words, the replication product is a copy of the template, implying that it can itself readily act as a template for both gene expression and DNA replication. Alternative approaches relying on genomic RNA and based on the Q β-replicase [3] or on a polymerase ribozyme [4], have been proposed. Despite recent improvement to minimize the production of undesired parasitic RNAs [5] and to design more replicable RNA templates [6], RNA replication by the Q β-replicase remains limited to RNAs with strong secondary structures, which is not suitable for expression of many protein-coding sequences. In contrast, the protein-primed Φ29 replication system is compatible with amplification of virtually any genes provided the linear template is flanked with the Φ29 replication origins. Newly engineered RNA polymerase ribozymes enable synthesis of long >100-nt RNA [7], including of RNAs with catalytic activity [8]. Further development toward self-replicating RNA protocells (RNA-based life) will however necessitate to expand template generality.

SUPPLEMENTARY TABLES & FIGURES

**Construct**	**Length (bp)**	**Origins of replication**	**Comments**
Terminal protein (TP, *p3*)	932	No	MW of protein:31 kDa
DNA polymerase (DNAP, *p2*)	2,000	No	MW of protein: 66 kDa
p5	508	No	MW of protein: 13.3 kDa
p6	447	No	MW of protein: 12 kDa
oriLR-p6	835	OriR194, OriL191	T7 terminator (T7t)
oriLR-p2-p3	3,212	OriR194, OriL191	tandem-VSV and T7t
oriL68-p2-p3	2,959	OriL68 at both ends	tandem-VSV and T7t
oriLR-p6-p5	1,382	OriR194, OriL191	tandem-VSV and T7t
TP-*oriLR-p2-p3*	3,212	Yes	parental TPs at 5'-ends
Φ29-genome	19,282	Yes	parental TPs at 5'-ends
p2-p3	-	No	tandem-VSV and T7t
p6-p5	-	No	tandem-VSV and T7t

Supplementary Table 1: List of the DNA constructs used in this study.

Primer	Sequence 5’ → 3’
73 ChD	GCGAAATTAATACGACTCACTATAGGGAGACC
91 ChD	AAAAAACCCCTCAAGACCCGTTTAGAGG
173 ChD	CACACAGGAAACAGCTATGAC
181 ChD	CAAAAAACCCCTCAAGACCCGTTTAGAGG
185 ChD	GAGACCACAACGGTTTCCCTCTAG
190 ChD	TTTTTTTTTTTTTTTTTTTTTTTTTTTTTTTATGCTAGCCCGGGGATATC
361 ChD	GCGAAATTAATACGACTCACTATAGGGAGACCACAACGGTTTCCCTCTAG
363 ChD	AAAGTAAGCCCCCACCCTCACATGATAGCGAAATTAATACGACTCACTATAGGGAGACC
364 ChD	AAAGTAGGGTACAGCGACAACATACACAAAAAACCCCTCAAGACCCGTTTAGAGG
365 ChD	CAGTCACGACGTTGTAAAACGAC
422 ChD	GGTCTCCCTATAGTGAGTCGTATTAGCAGTCGACGGGCCCGGGATCCGAT
423 ChD	ATCGGATCCCGGGCCCGTCGACTGCTAATACGACTCACTATAGGGAGACC
430 ChD	CCATACAGGCTGTTCAGCATC
431 ChD	GTGTCATCGACCAGCACAAC
432 ChD	CCATGGATTCTTCCAGGGTG
433 ChD	CACCCTGGAAGAATCCATGG
434 ChD	AAAGTAAGCCCCCACCCTCACATGATAG
435 ChD	AAAGTAGGGTACAGCGACAACATACAC
491 ChD	AAAGTAAGCCCCCACCCTCACATG
492 ChD	AAAGTAGGGTACAGCGACAACATACAC

Supplementary Table 2: List and sequences of the primers used in this study.

Primer	FW/RV	Purpose	Comments
73 ChD	FW	include T7 promoter	7 nt before T7 promoter
91 ChD	RV	include T7 terminator	5’-AAA is potential ori
173 ChD	RV	isolate DNAP gene from pUC57	
181 ChD	RV	include T7 terminator	5’-CAA, no possible origin
185 ChD	FW	remove T7 promoter	
190 ChD	RV	add poly-A tail	
361 ChD	FW	introduce T7 promoter	
363 ChD	FW	extend T7 promoter with minimal ori oriL24,	5’-phosphorylated
364 ChD	RV	extend T7 terminator with minimal ori oriR27,	5’-phosphorylated
365 ChD	FW	isolate DNAP gene from pUC57	
422 ChD	FW	assembly PCR DNAP-TP	
423 ChD	RV	assembly PCR DNAP-TP	
430 ChD	RV	for sequencing of DNAP-TP	
431 ChD	RV	for sequencing of DNAP-TP	
432 ChD	RV	for sequencing of DNAP-TP	
433 ChD	FW	for sequencing of DNAP-TP	
434 ChD	FW	assembly PCR	5’-phosphorylated
435 ChD	RV	assembly PCR	5’-phosphorylated
491 ChD	FW	oriL24, for all oriL68 and oriL191	5’-phosphorylated
492 ChD	RV	oriR25, for all oriR194	5’-phosphorylated

Supplementary Table 3: Additional information on the primers used in this study.

Sequence 5’ → 3’
AAAGTAAGCCCCCACCCTCACATGATACCATTCTCCTAATATCGACATAATCCGTCGATCCTCGG
CATACCATGATCAGGGAGGGAAACTACTACTTAATATATCAATCTATAGACCTACTAGATAGGTTT
GTCAATGAACAACATAAAACGACACAGAATCCCACGTTTTAGCGCTTCGTCTGTGTCGCATGTGA
AATTAATACGACTCACTATAGGGAGACCACAACGGTTTCCCTCTAGAAATAATTTTGTTTAACTTTA
AGAAGGAGATATACATATGCCGCGTAAAATGTACAGCTGCGATTTTGAAACGACGACGAAAGTTG
AAGATTGCCGTGTCTGGGCCTATGGTTATATGAACATCGAAGACCATTCAGAATATAAAATTGGCA
ACTCGCTGGATGAATTTATGGCGTGGGTGCTGAAAGTTCAGGCCGACCTGTACTTCCACAATCTG
AAATTTGATGGTGCGTTCATTATCAACTGGCTGGAACGTAATGGCTTTAAATGGAGCGCCGATGG
TCTGCCGAACACCTATAATACGATTATCTCTCGTATGGGCCAATGGTATATGATTGATATCTGCCT
GGGCTACAAAGGTAAACGCAAAATTCATACCGTGATCTATGACAGCCTGAAAAAACTGCCGTTTC
CGGTGAAGAAAATTGCGAAAGATTTCAAACTGACCGTCCTGAAAGGCGATATTGACTATCACAAA
GAACGTCCGGTTGGTTACAAAATCACGCCGGAAGAATATGCGTACATTAAAAACGATATCCAGAT
TATCGCAGAAGCTCTGCTGATTCAGTTTAAACAAGGCCTGGATCGCATGACCGCCGGCAGTGACT
CCCTGAAAGGTTTCAAAGATATCATCACCACGAAAAAATTTAAGAAAGTGTTCCCGACCCTGAGC
CTGGGTCTGGATAAAGAAGTTCGTTATGCATACCGCGGCGGTTTTACGTGGCTGAACGACCGTTT
CAAAGAAAAAGAAATTGGCGAGGGTATGGTCTTTGATGTGAATAGTCTGTATCCGGCTCAGATGT
ACTCCCGCCTGCTGCCGTATGGCGAACCGATCGTTTTCGAGGGTAAATATGTCTGGGATGAAGA
CTACCCGCTGCATATTCAGCACATCCGTTGTGAATTTGAACTGAAAGAAGGCTATATTCCGACCAT
TCAAATCAAACGTAGCCGCTTCTATAAGGGTAACGAATACCTGAAAAGCTCTGGCGGTGAAATCG
CAGACCTGTGGCTGAGTAACGTCGATCTGGAACTGATGAAAGAACATTACGATCTGTACAACGTT
GAATACATCTCCGGCCTGAAATTTAAAGCCACCACGGGTCTGTTTAAAGATTTCATTGACAAATGG
ACCTACATCAAAACCACGTCTGAAGGTGCAATCAAACAGCTGGCTAAACTGATGCTGAACAGCCT
GTATGGCAAATTTGCATCTAATCCGGATGTTACCGGTAAAGTCCCGTACCTGAAAGAAAATGGCG
CTCTGGGTTTTCGCCTGGGCGAAGAAGAAACCAAAGATCCGGTGTATACGCCGATGGGTGTTTT
CATTACCGCGTGGGCACGTTACACCACCATCACCGCCGCACAAGCGTGCTATGACCGCATTATC
TACTGTGATACCGACTCAATTCATCTGACCGGCACGGAAATCCCGGATGTGATTAAAGATATCGT
TGACCCGAAAAAACTGGGTTATTGGGCACACGAATCGACCTTTAAACGTGCTAAATACCTGCGCC
AGAAAACGTACATCCAAGACATCTACATGAAAGAAGTCGATGGCAAACTGGTGGAAGGTTCACCG
GATGACTATACCGACATTAAATTTTCGGTGAAATGCGCCGGCATGACCGATAAAATTAAGAAAGAA
GTGACGTTCGAAAATTTCAAAGTGGGTTTCAGTCGCAAAATGAAACCGAAACCGGTCCAAGTTCC
GGGCGGCGTTGTGCTGGTCGATGACACCTTCACGATCAAATAAGAATTGTACTAGAGTATCTGTT
AGTTTTTTTCTACTAGAGTACTAGAGTATCTGTTAGTTTTTTTCATCGGATCCCGGGCCCGTCGAC
TGCTAATACGACTCACTATAGGGAGACCACAACGGTTTCCCTCTAGAAATAATTTTGTTTAACTTTA
AGAAGGAGATATACATATGGCACGCAGCCCGCGCATCCGCATCAAAGATAACGACAAAGCCGAA
TACGCCCGCCTGGTGAAAAATACGAAAGCTAAAATCGCACGTACCAAGAAAAAATATGGCGTGGA
TCTGACGGCTGAAATTGACATCCCGGATCTGGACTCATTTGAAACCCGCGCGCAGTTCAATAAAT
GGAAAGAACAAGCTAGCTCTTTTACGAACCGTGCGAATATGCGCTATCAGTTCGAGAAAAACGCC
TACGGTGTGGTTGCATCGAAAGCTAAAATTGCGGAAATCGAACGTAACACCAAAGAAGTTCAACG
CCTGGTCGATGAAAAAATCAAAGCCATGAAAGACAAAGAATATTACGCAGGCGGTAAACCGCAGG
GCACGATTGAACAACGTATCGCCATGACCTCACCGGCACATGTGACGGGTATCAACCGTCCGCA
CGATTTTGACTTCAGTAAAGTTCGCAGTTACTCCCGTCTGCGCACCCTGGAAGAATCCATGGAAA
TGCGCACGGATCCGCAGTATTACGAAAAGAAAATGATTCAGCTGCAACTGAATTTTATCAAAAGC
GTCGAAGGCTCATTTAACTCGTTCGATGCGGCCGACGAACTGATTGAAGAACTGAAGAAAATTCC
GCCGGATGACTTTTATGAACTGTTCCTGCGTATTAGCGAAATCTCTTTTGAAGAATTCGATTCTGA
AGGCAACACCGTCGAAAATGTGGAGGGTAACGTTTACAAAATTCTGTCGTATCTGGAACAATATC
GTCGTGGTGATTTTGATCTGTCGCTGAAAGGCTTCTAATAGCATAACCCCTTGGGGCCTCTAAAC
GGGTCTTGAGGGGTTTTTTGCCTCCTATGATTGGTTGTCTTATTACCTTACTTCTATTATAGTATAA
CATGTTAAACGATAGTTTGTCTACCCTTTTCGACAAATTGATGATAATAAATAGTATAGGTATATAG
TCGTGATTTAGTTGTTAGATTCTTGTCGAAGATAGTCGGTCAATGGGGAAATGGTGTATGTTGTCG
CTGTACCCTACTTT

Supplementary Table 4: Sequence of the *oriLR-p2-p3* construct.

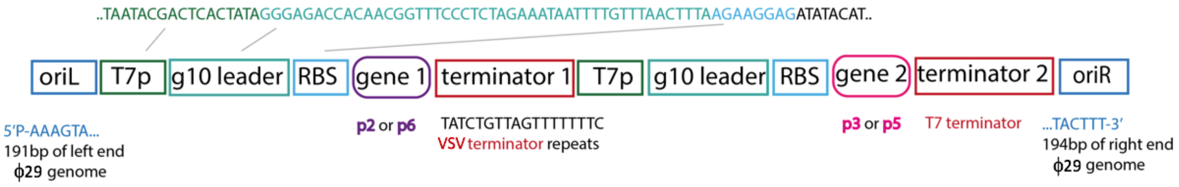

Supplementary Figure 1: Schematic of the design of the two-gene constructs and sequences of key regulatory elements. The *p2* and *p3* genes, and the *p6* and *p5* genes were assembled in two-cistron transcription units as illustrated in Fig. 1C. Constructs that do not contain the left and right ends of the Φ29 genome (origins of replication) were also assembled. The complete sequence of the *oriLR-p2-p3* construct is reported in Supplementary Table 4.

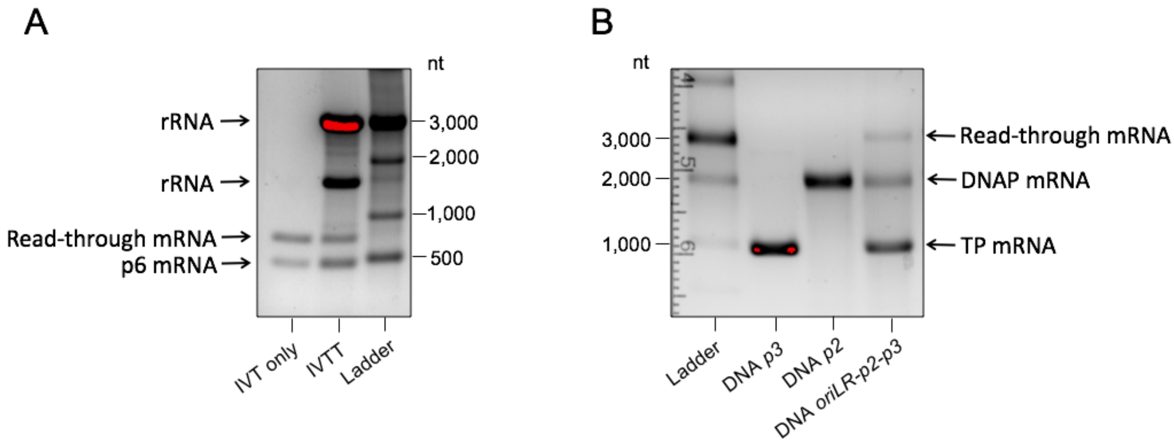

Supplementary Figure 2: Visualization of transcription activity and production of read-through RNA products. (A) The termination efficiency of the T7 terminator was determined by transcribing the *oriLR-p6* template in a transcription-only (IVT) system (the *in vitro* T7 Ribomax Large Scale RNA production kit, Promega) or in PURE*frex* (IVTT). Reading through the T7 terminator extends the transcript size from ~400 to ~600 nt, corresponding to the appended *oriR* sequence. Based on band intensity analysis (and assuming that band intensity linearly scales with the total amount of RNA present), an estimation of the termination efficiency is 70% in PURE*frex* and 56% in the T7 RNA production kit. (B) The efficiency of the VSV-repeat sequence to terminate transcription was assessed using the *oriLR-p2-p3* construct. PURE*frex* without ribosome (Δribo) was used to avoid the 1.5-kb and 2.9-kb ribosomal RNAs (see gel in A) to interfere with the detection of the similarly-sized transcripts. Quantitation of the band intensities for the three RNA species, namely the p2, p3 and p2-p3 read-through transcripts led to ~80% termination efficiency of the VSV repeat. The separate transcription activity of the *p2* and *p3* single-gene constructs is shown as a reference. We noticed that transcription of the *oriLR-p2-p3* template leads to more mRNA from the *p3* gene than from *p2* although they are both under control of the same promoter (lane 4). A possible explanation is that the occurrence of transcription initiation of the second gene (*p3*) is higher because its promoter is in close proximity from the upstream terminator of the first gene (*p2*). As a consequence, the dropped off RNA polymerase could rebind to the second promoter increasing the probability of complex formation compared to that for the first promoter. Transcription assays with dual-gene constructs with different spacing between the two transcription units should be conducted to confirm this hypothesis.

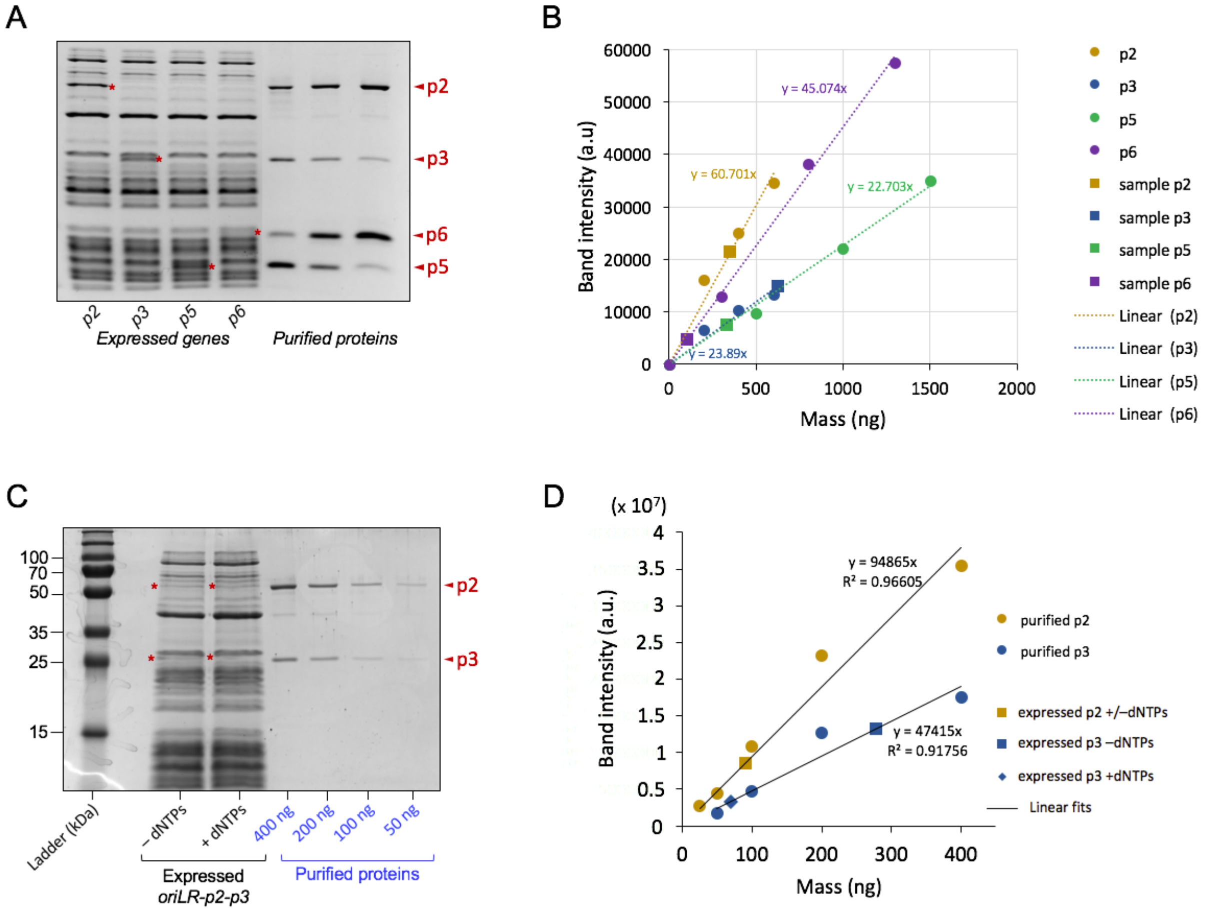

Supplementary Figure 3: Estimation of the concentrations of the synthesized proteins. The amounts of Φ29 DNA replication proteins synthesized in PURE*frex* (4 h expression at 30 °C) were estimated by band intensity analysis on a coommassie stained protein gel (12% polyacrylamide). (A) The proteins were expressed separately from their monocistronic DNA templates (~3 nM) to determine an upper limit of protein expression levels. The asterisks indicate the synthesized proteins. As references, the purified Φ29 proteins were loaded at three different known concentrations. (B) Standard curves representing the band intensity values for the purified proteins of known concentrations (circle symbols) were measured from the gel shown in (A). Linear fits were calculated and displayed as dashed lines. The band intensity of the expressed protein (square symbols) was appended on the linear fit. The corresponding mass was calculated and converted into concentration. Linearity of the calibration curves down to 25 ng protein was confirmed in other experiments. The estimated concentrations are 1.0 µM for p2 (DNAP), 4.0 µM for p3 (TP), 5.0 µM for p5 (SSB) and 1.7 µM for p6 (DSB). Compared to the amounts of proteins used in the *in vitro* Φ29 DNA amplification system [9], this is around 60 times higher for the p2 and p3, and 5 times lower for the p5 and p6 proteins. Therefore, an excess of the *p5* and *p6* templates with respect to the *p2* and *p3* genes was used in replication experiments. (C) The *oriLR-p2-p3* construct (2.5 nM) was expressed with or without dNTPs and the amount of synthesized p2 and p3 proteins was estimated by PAGE. Purified p2 and p3 were loaded on the same gel at four to five different concentrations for calibration. (D) Standard curves and concentrations of expressed p2 and p3 were determined as described in (B). Under these conditions, ~100 nM of p2 (with or without

dNTPs), and ~700 nM (–dNTPs) or ~180 nM (+dNTPs) of p3 were measured. The lower concentration of p3 detected in the presence of dNTPs may be assigned to its depletion by DNA linkage upon replication initiation. Although these concentrations are significantly reduced compared to IVTT from single-gene DNA templates (B), they exceed the amounts that are sufficient for *in vitro* amplification of heterologous DNA, i.e. 16 nM for p2 and 65 nM for p3 [9]. Moreover, the upper limit of amplified DNA concentration with an excess of dNTPs is imposed by the concentration of p3, here ~700 nM, which is sufficient to amplify DNA by a factor ~140. Future works should aim at reducing the amount of synthesized p3 to limit replication of DNA to a level that is adequate for functional transmission of the genetic information upon division.

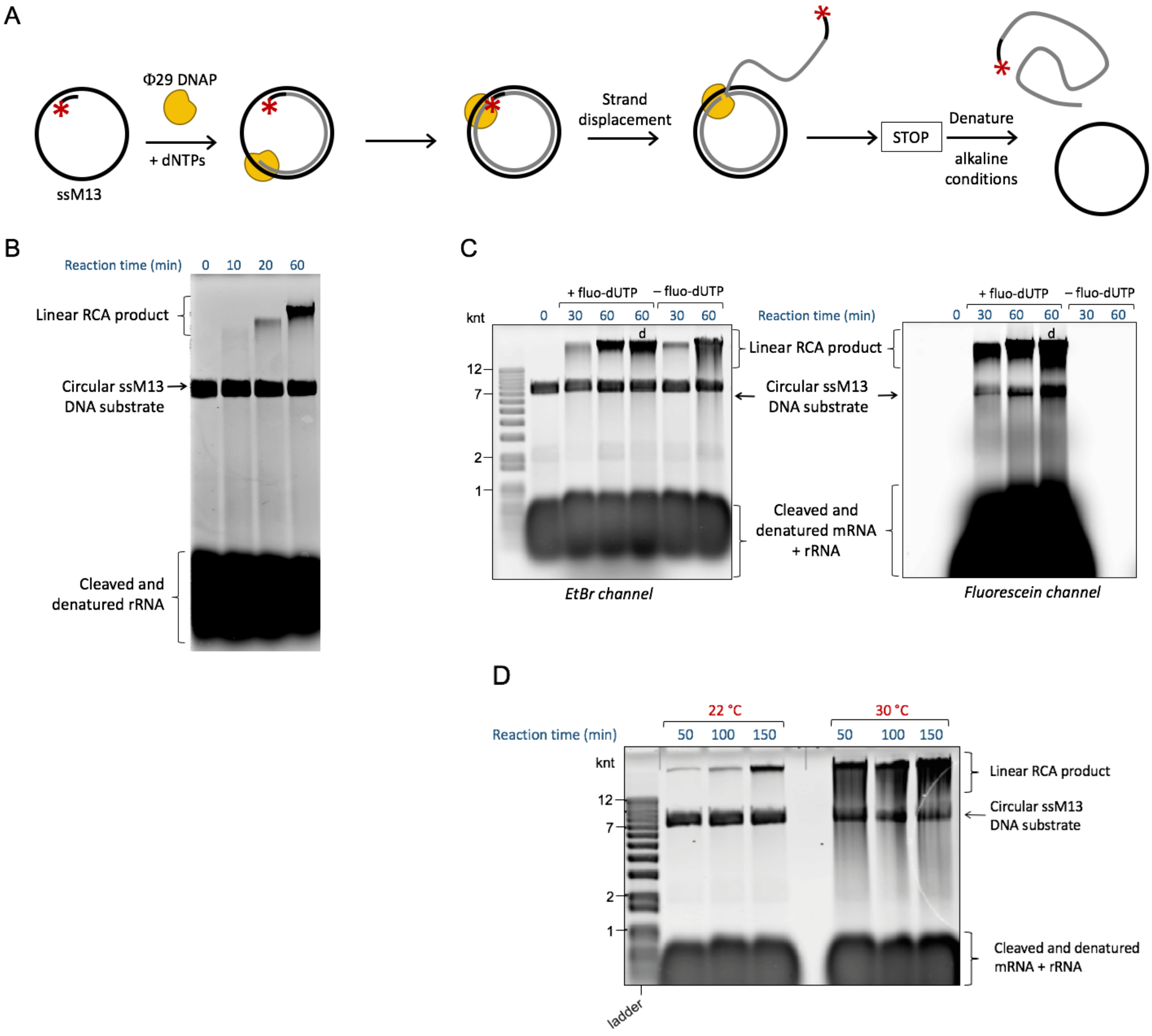

Supplementary Figure 4: Validation of the Φ29 DNAP activity by performing rolling circle amplification (RCA) assays. (A) Schematic of the RCA assay by the DNAP having a strand-displacement activity. A fluorescently labelled primer is hybridized on the circular ssM13 DNA to create a primer-template junction where the DNAP can initiate polymerization. Alternatively, a fluorescent dUTP analogue was used to visualize the newly synthesized DNA. Upon completion of one circle, the DNAP displaces the primer-strand and continues polymerization until the reaction is quenched. The denatured DNA is then imaged on an 0.7% alkaline agarose gel through EtBr (total nucleic acids) or through the fluorescent dye (elongated primer only). (B) A control experiment is performed with the purified DNAP supplemented in PURE*frex*. The circular ssM13 DNA substrate (7.4 knt) is visible, as well as the longer linear DNA products generated in the course of the reaction. (C) RCA by the DNAP synthesized in PURE*frex* from its *p2* gene. ‘d’ means that the samples were denatured at 95 °C. (D) Combined gene expression and RCA was demonstrated at 22 °C and 30 °C, the latter temperature yielding to a larger amount of *de novo* DNA.

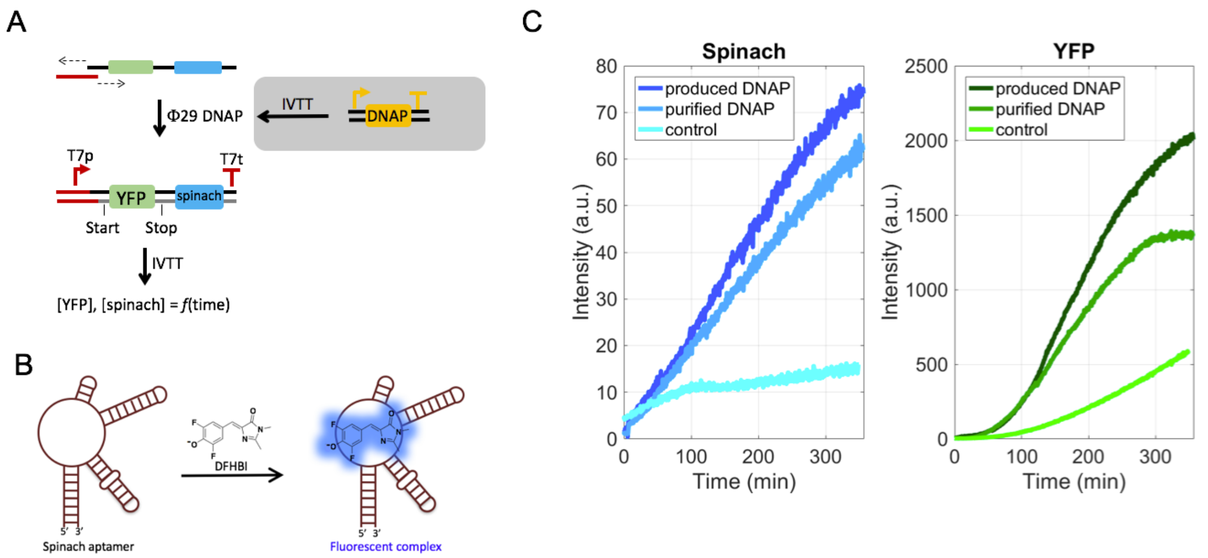

Supplementary Figure 5: The synthesized DNAP can elongate a primer-template junction and generate transcriptionally active dsDNA. (A) A T7 promoter-containing ssDNA oligonucleotide is hybridized on the *mYFPco-LL-Spi-T7t* ssDNA (Supplementary Methods) which serves as a template for the DNAP synthesized in PURE*frex* from the *p2* gene (no origins of replication). DNAP activity yields to transcriptionally active dsDNA. Transcriptional and translational activities is monitored through the Spinach and YFP fluorescence signals, respectively [10,11]. (B) Schematic of the Spinach technology, whereby binding of the DFHBI chromophore to the Spinach aptamer leads to formation of a fluorescent complex [12]. (C) Simultaneous detection of the Spinach and YFP fluorescence signals reporting the kinetics of synthesized mRNA and protein in real-time. As a positive control, the purified DNAP is used. Interestingly, no significant time delay is observed in the Spinach kinetics, indicating that within a few minutes only, enough DNAP is synthesized to produce transcriptionally active dsDNA at high transcription rate. Faithful copying of DNA is assessed by the functional production of the encoded YFP protein. The residual production of RNA and protein in the negative control sample that did not contain the *p2* gene (only the oligo-bound ssDNA) very likely comes from carryover of the dsDNA from the asymmetric PCR. a.u., arbitrary units.

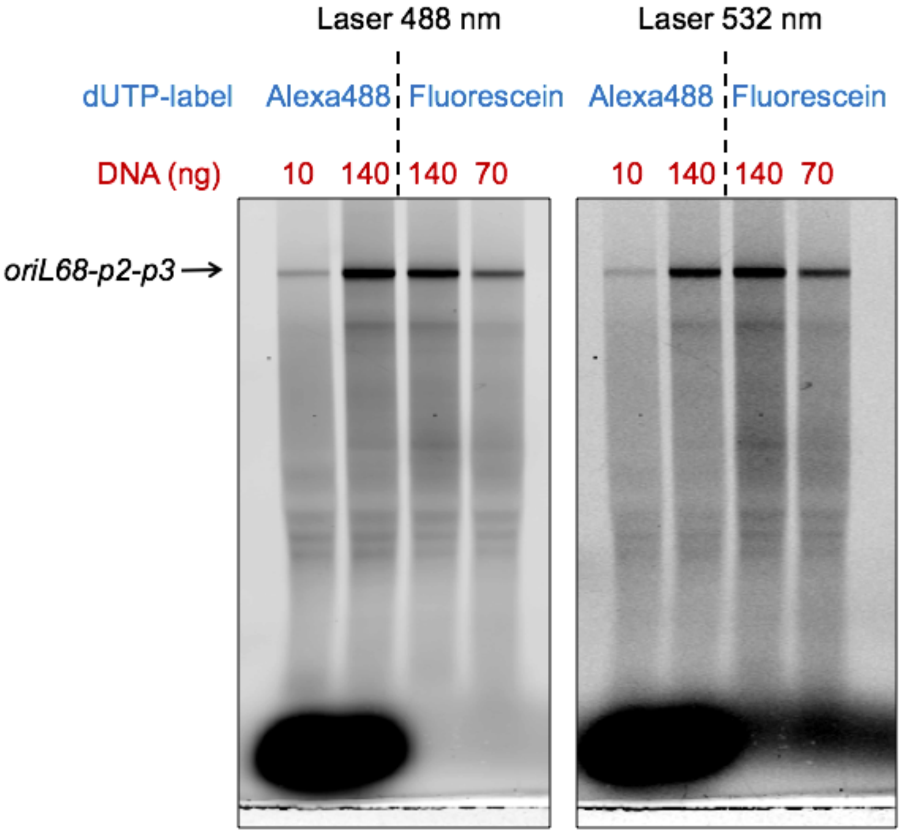

Supplementary Figure 6: Incorporation of fluorescent nucleotides by the synthesized DNAP in protein-primed DNA replication reactions. PURE*frex* solution was supplemented with the *oriL68-p2-p3* template, 20 mM NH_4SO_2, dNTPs and a fluorescent analogue of dUTP (either dUTP-Alexa488 or dUTP-fluorescein) to visualize the synthesized DNA. The different amounts of input DNA templates are indicated. The amount of incorporated nucleotides increases upon increasing the concentration of template DNA in the range of 10 – 140 ng (i.e. 0.3 – 3.8 nM).

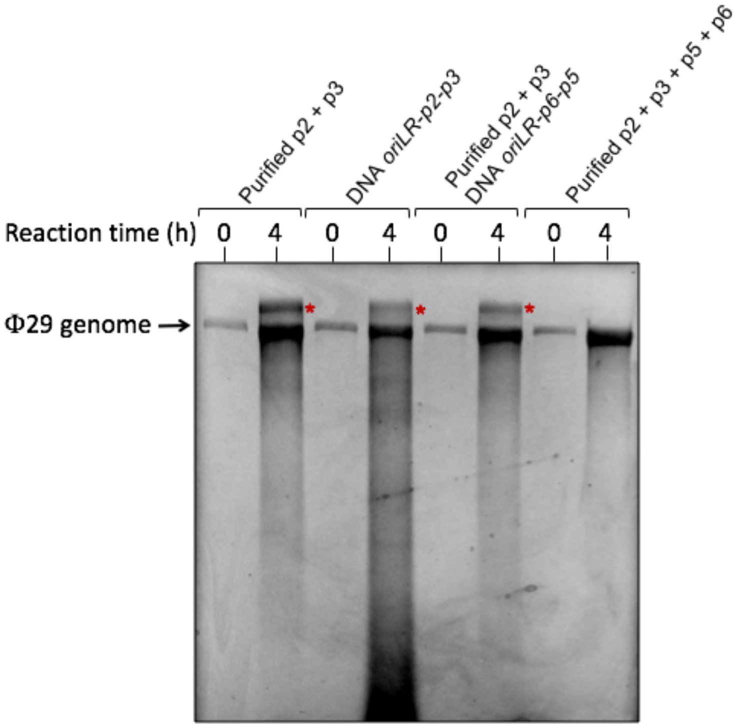

Supplementary Figure 7: Amplification of the Φ29 genome in the PURE system by the purified and/or expressed Φ29 DNA replication proteins. The experimental conditions are reported in Supplementary Methods. The input and amplification products were visualized on an alkaline agarose gel. All samples show replication of the Φ29 genome after 4 h. The sample with all four purified proteins has less smear and no upper band (labelled with an asterisk), indicating optimized protein concentrations. The large smear with synthesized p2 and p3 from expression of the *oriLR-p2-p3* DNA suggests a too high concentration of the DNAP that may initiate on strand-displaced ssDNA in the absence of p5. The upper band (marked with an asterisk) is only visible when high amounts of Φ29 genome are loaded on gel and it does not appear in the presence of purified or synthesized (Fig.2B, right panel) p5 and p6 proteins. A possible cause is the tendency of TP (that resisted protease digestion) to aggregate in the gel or to the formation of genome dimers.

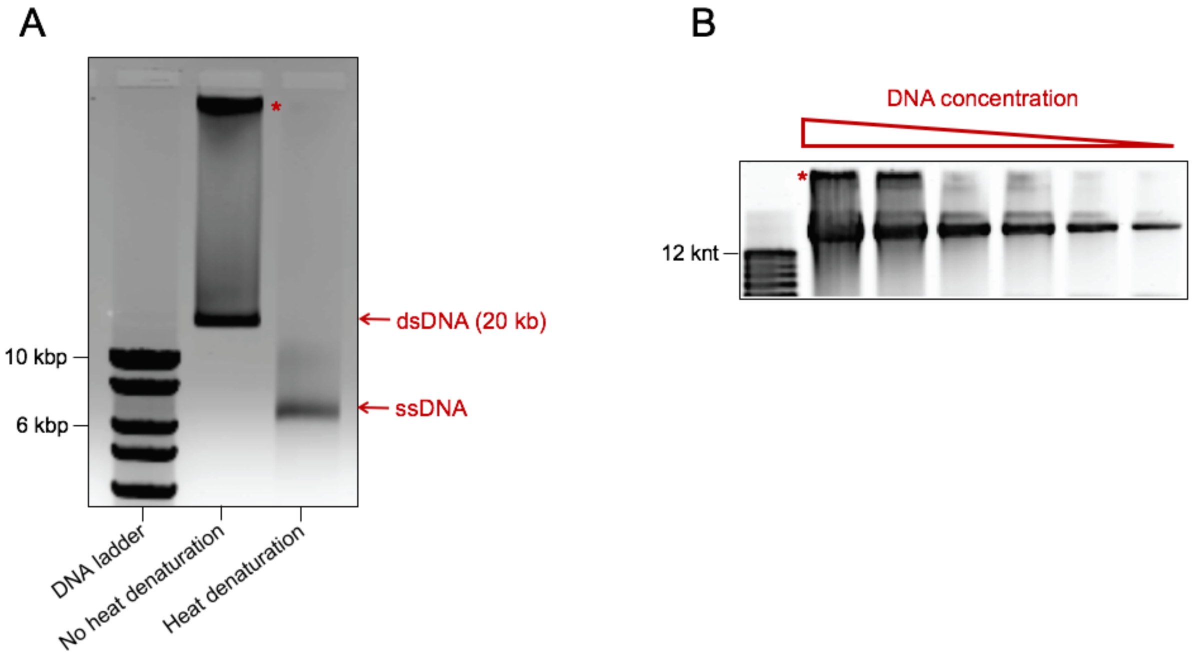

Supplementary Figure 8: Characterization of the Φ29 genome on neutral and alkaline agarose gels. (A) Neutral agarose gel with 50 ng of the Φ29 genome DNA in the presence of 0.1% SDS, with or without heat denaturation at 95 °C for 2.5 min. (B) The Φ29 genome DNA was loaded on a 0.7% alkaline agarose gel at different concentrations, from 300 ng (left) to 10 ng (right-most lane). Note the presence of an upper band (indicated by the asterisk) in both gels as already reported in Fig. 2B and Supplementary Fig. 7. After heat denaturation of the Φ29 genome, only one band of ssDNA is visible. This indicates that the upper band corresponds to the Φ29 genome present in a different state or folded structure.

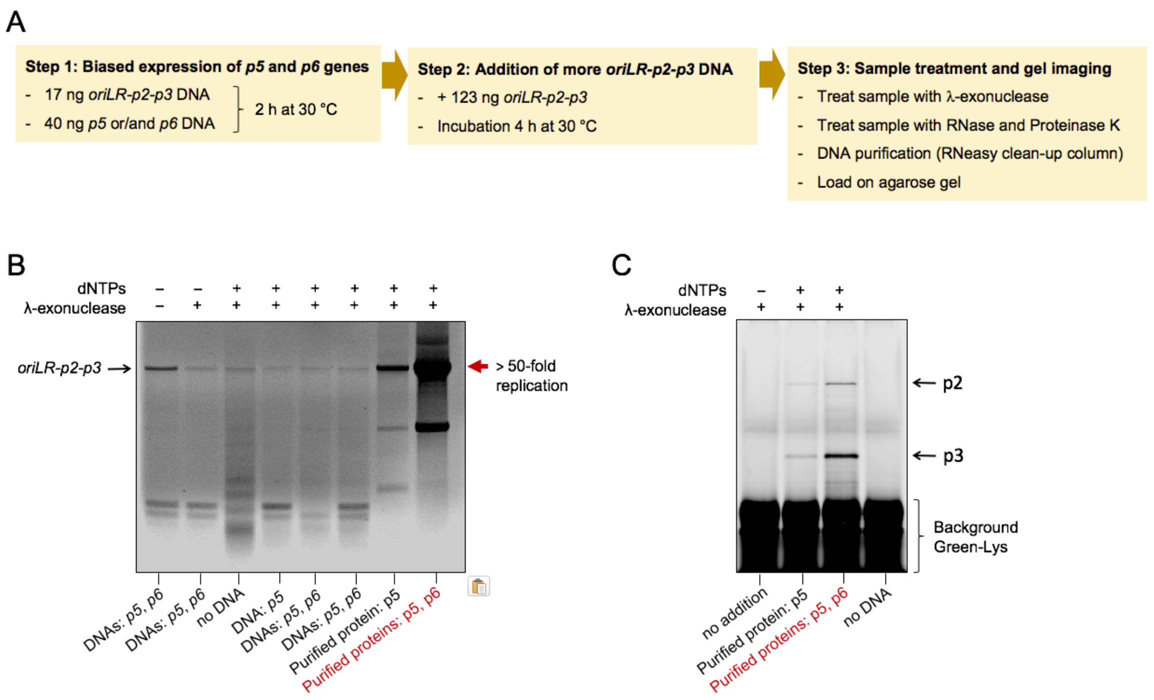

Supplementary Figure 9: Amplification of the *oriLR-p2-p3* DNA in PURE*frex* is enhanced by the purified p5 and p6 proteins. (A) Workflow of experiments. The p5 and p6 were supplied either from the start as purified proteins (7.5 µg p5 and/or 1.5 µg p6) or were expressed *in situ* from their genes (no *ori* sequences). To bias gene expression towards the p5 or/and p6 proteins, the first 2 h of reaction were incubated at 30 °C with a low amount of the *oriLR-p2-p3* construct (17 ng in a 20-µL reaction, i.e. 0.4 nM) compared to that of the *p5* and/or *p6* DNAs (40 ng each, i.e. 6.4 nM *p5* and 7.2 nM *p6*). Then, 123 ng (3.1 nM) of the *oriLR-p2-p3* template was added to all samples to obtain enough of the input DNA for the replication reaction. The samples were further incubated for 4 h at 30 °C, prior to λ-exonuclease treatment (1 µL in 10 µL, incubation 40 min at 30 °C) and DNA purification. The samples were finally imaged on a 1.1% neutral agarose gel stained with EtBr (B). (B) Clear amplification is only visible for samples supplied with purified proteins. Co-expression of the *p5* and/or *p6* genes does not produce sufficient amounts of proteins to achieve significant amplification. This two-step gene expression protocol was repeated and led to similar results. A lower amplification band was observed between 1 kbp and 1.5 kbp (see also Fig. 3B, Supplementary Fig. 10, 12B, 13A). It is known that the Φ29 DNA replication system can potentially generate a variety of short DNA products in vitro [13]. Their formation may involve a template-switching mechanism of the DNA polymerase. The produced short DNA molecules can then be replicated to generate short replicons. Alternatively, abortion of DNA replication on a specific sequence or when the polymerase encounters a protein roadblock may cause formation of the observed short DNA product. Efficient replication mediated by the purified p5 and p6 proteins also leads to a band above the full-length product (see also Fig. 3B). Although we do not know the exact nature of this DNA product and the mechanism that generates it, we presume it originates from some interwoven dsDNA and ssDNA due to uncompleted replication, from DNA aggregation via TP that resisted protease digestion or from a distinct long-sized replication product whose nature remains to be elucidated. (C)

Purified DNA from (B) was subsequently used as template for a second IVTT reaction in PURE*frex* supplemented with GreenLys. The p2 and p3 translation products were analysed by PAGE. The expected protein pattern was generated when purified proteins were supplied.

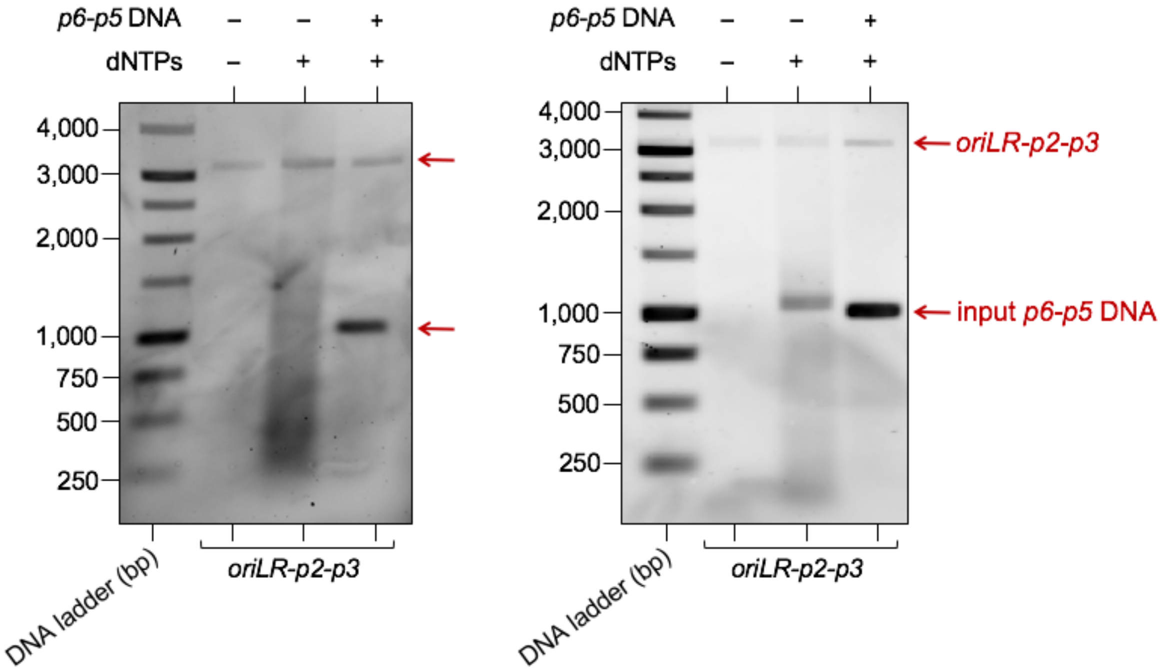

Supplementary Figure 10: Co-expression of the *p6-p5* DNA fails to improve replication efficiency of the *oriLR-p2-p3* template. The amplification products from two independent experiments are shown on separate agarose gels. 6.9 nM of *p6-p5* DNA and ~1.3 nM (left) or ~1.5 nM (right) of *oriLR-p2-p3* were used. No obvious enhancement of the amplification yield of the *oriLR-p2-p3* template upon co-expression of the *p6-p5* DNA can be seen. Possible origins of the lower replication band are reported in the legend of Supplementary Fig. 9B.

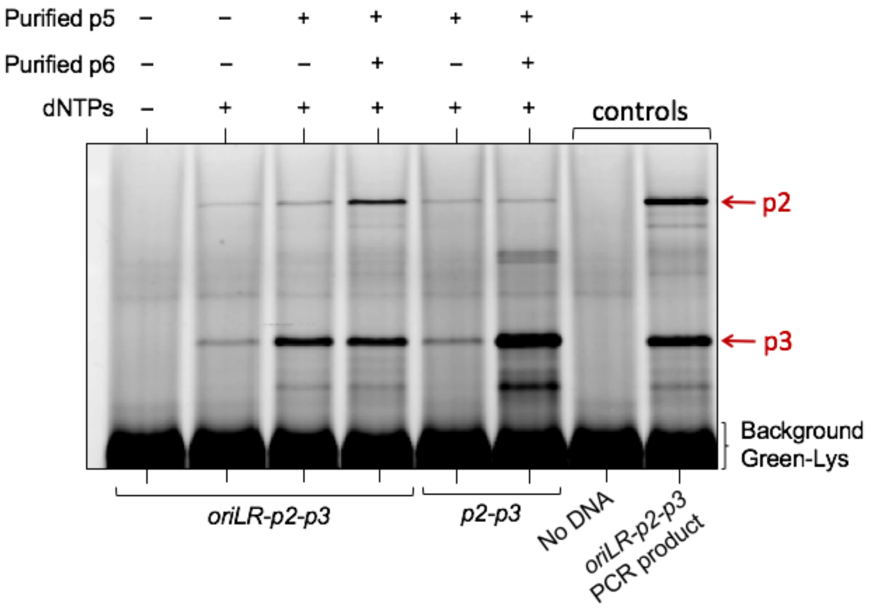

Supplementary Figure 11: The newly synthesized DNA is able to generate the encoded p2 and p3 proteins in PURE*frex*. PAGE analysis of the PURE*frex* translation products when using as input template the replication product of the *oriLR-p2-p3* or *p2-p3* DNA template. Addition of the purified p5 and p6 for DNA replication led to a pattern of p2 and p3 proteins resembling that of the positive control.

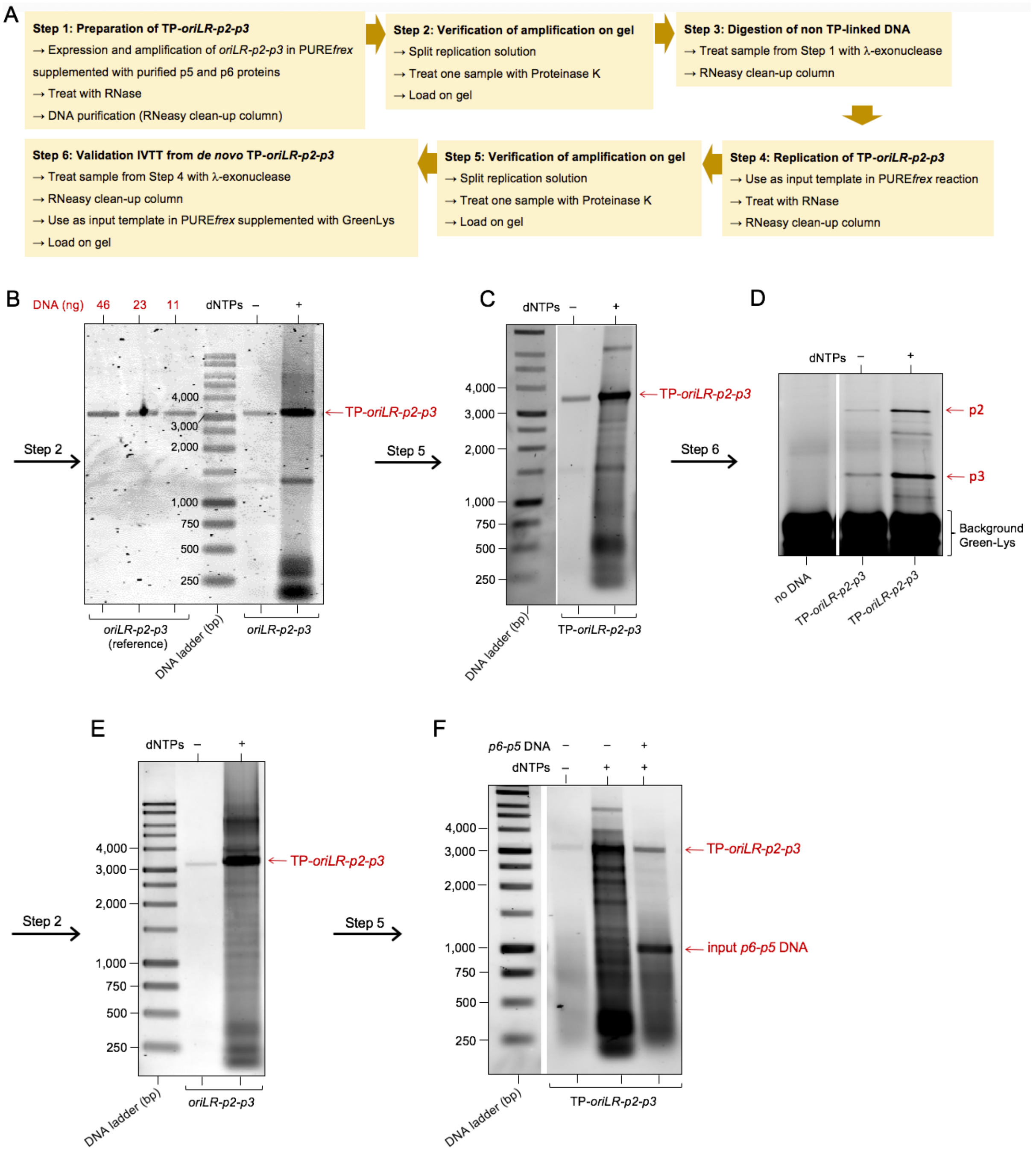

Supplementary Figure 12: TP-capped DNA is effectively replicated and the newly synthesized DNA can be transcribed and translated after two successive IVTTR cycles. (A) Workflow of the experiments. Starting from the *oriLR-p2-p3* template, a first IVTTR cycle produces TP-*oriLR-p2-p3* that is purified and fed into a new IVTTR reaction. The second-generation TP-*oriLR-p2-p3* DNA is finally transcribed and translated in a third PURE*frex* reaction. See also a schematic of the cycles in Fig. 4E. (B) Replication products starting with the *oriLR-p2-p3* template. Varying amounts of purified *oriLR-p2-p3* DNA were loaded on the same gel to estimate the concentration of TP-*oriLR-p2-p3*. (C) Replication products starting with the TP-*oriLR-p2-p3* template. (D) PAGE analysis of the IVTT products labelled with GreenLys using the amplified TP-*oriLR-p2-p3* DNA as input template. (E, F) Second independent experiment showing the gels of Step 2 (E) and Step 5 (F). Here, coupled

expression-replication of the TP-*oriLR-p2-p3* template was also conducted in the presence of the *p6-p5* DNA (no *ori* sequences). Clear amplification of the input TP-capped DNA and reduction of side products can be observed.

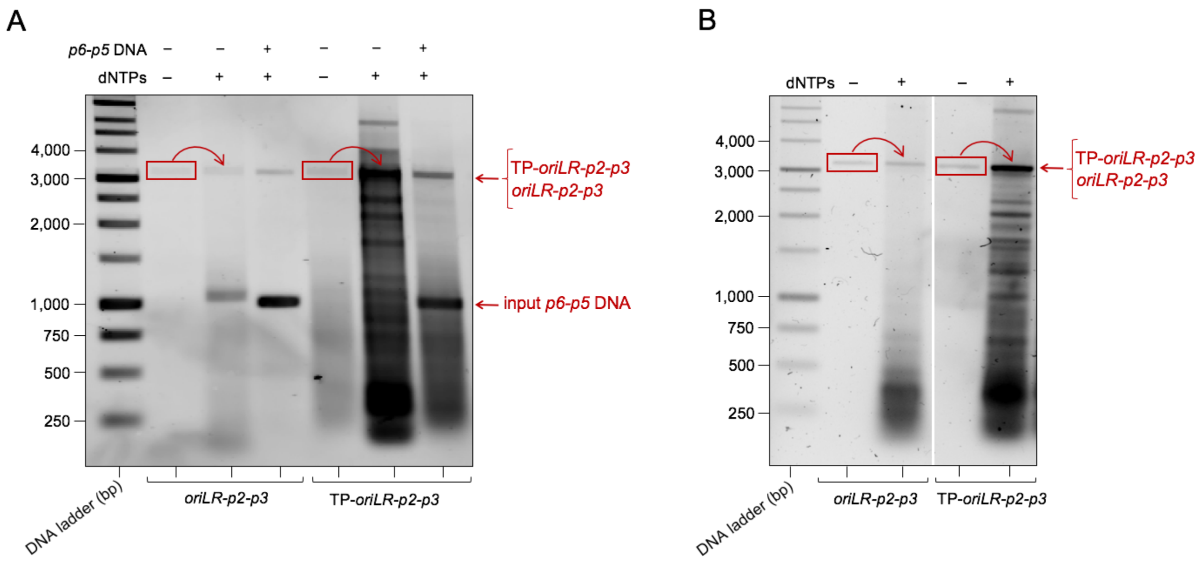

Supplementary Figure 13: Direct comparison of amplification efficiency between the TP-capped and TP-uncapped replication substrates. All experiments with TP-bound DNA were conducted in parallel to reactions with uncapped DNA and the end-point replication solutions were loaded on the same gel for comparison. The outcome of two independent experiments is shown in (A) and (B). (A) The lanes with the TP-*oriLR-p2-p3* template were shown in Supplementary Fig. 12F, while the lanes with the *oriLR-p2-p3* template were already displayed in Supplementary Fig. 10. About 1.5 nM of either the *oriLR-p2-p3* or the TP-*oriLR-p2-p3* DNA was used. (B) The part of gel with the TP-*oriLR-p2-p3* template was shown in Fig. 4B. About 1.9 nM of either the *oriLR-p2-p3* or the TP-*oriLR-p2-p3* DNA was used. It can be concluded that starting from a similar amount of DNA, only the TP-capped substrate is notably amplified both in the absence of *p6-p5* (~10-fold vs. ~2-fold without TP) and in its presence (~4-fold increase from TP-capped DNA).

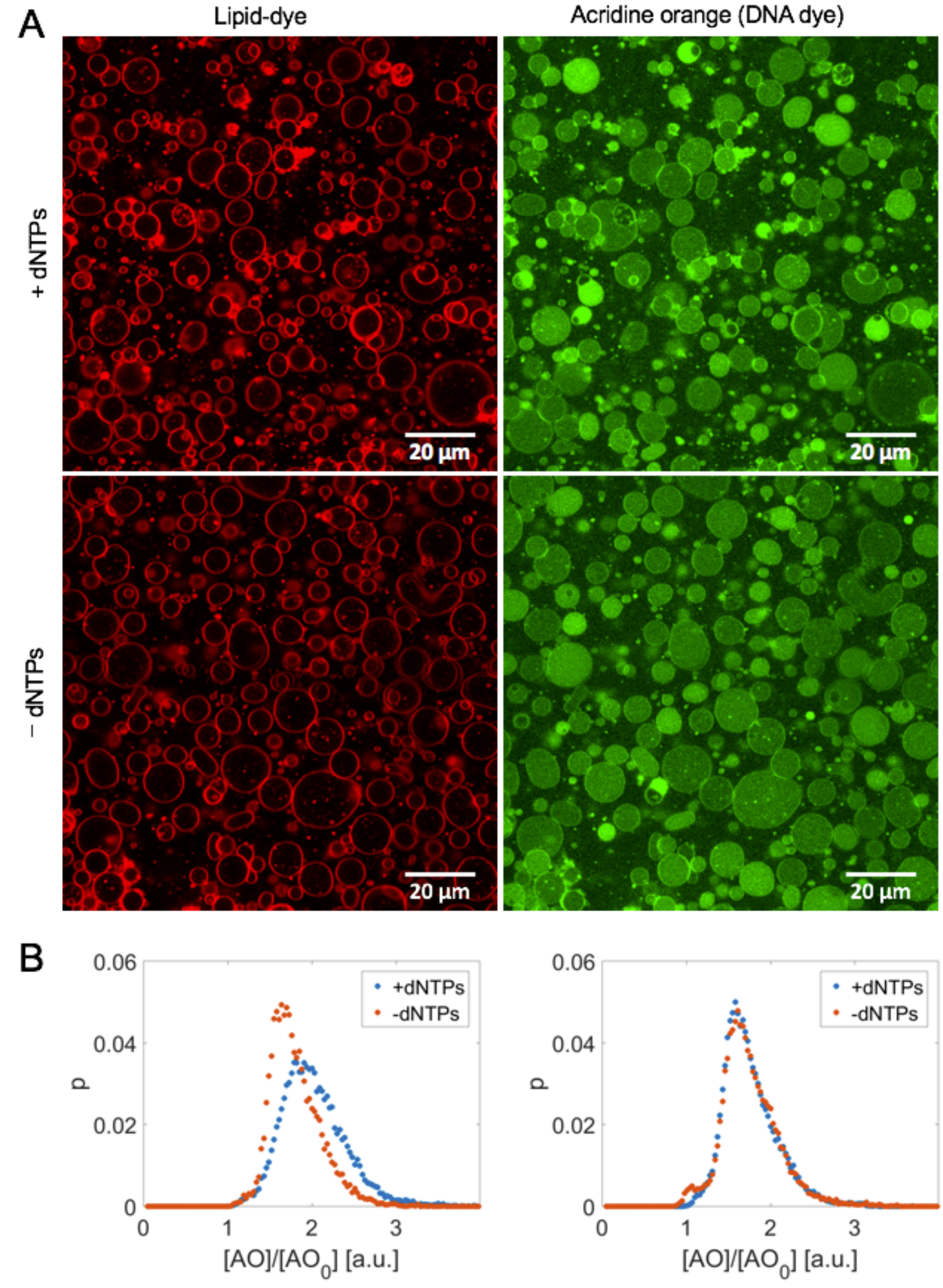

Supplementary Figure 14: Co-expression of *TP-oriLR-p2-p3* and *p6-p5* templates inside liposome fails to produce detectable amount of synthesized DNA. (A) Fluorescence confocal microscopy images of liposomes. Membrane dye and acridine orange signals are shown in red and green, respectively. The top images correspond to a replication sample, while the bottom images correspond to a control sample, where the dNTP mix was omitted. (B) Histograms of the acridine orange lumen intensity normalized to background (selected extraliposomal regions of interest) for two independent experiments, one starting from 1.3 µL TP-*oriLR-p2-p3* DNA (left; 21,463 and 13,080 liposomes were analysed for the +dNTPs and –dNTPs conditions, respectively), the other with 2.5 µL (right; 34,853 and 31,612 liposomes were analyzed for the +dNTPs and –dNTPs conditions, respectively). No substantial increase of the DNA dye intensity upon replication (+dNTPs) can be observed.

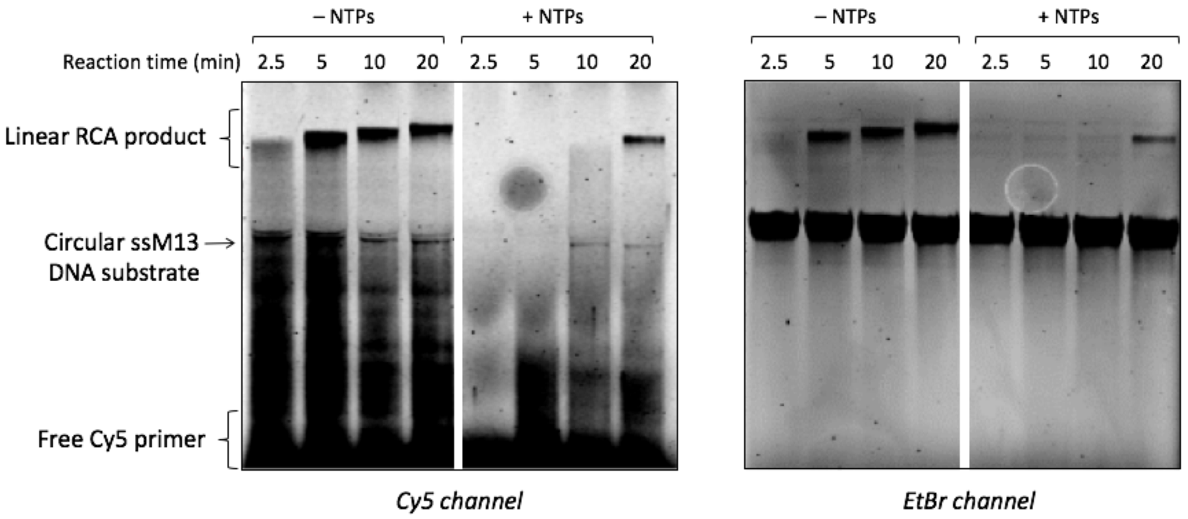

Supplementary Figure 15: Rolling circle amplification (RCA) with the Φ29 DNAP is slower in the presence of ribonucleotides (NTPs). RCA experiments were performed according to the reaction scheme shown in Supplementary Fig. 4A using the Cy5-primed ssM13 template. The commercial Φ29 DNAP and accompanying buffer were used as described in Supplementary Materials and Methods. The +NTPs sample contains 3 mM of both ATP and GTP, and 1 mM of CTP and UTP as present in the PURE system. The concentration of each dNTP was 0.3 mM. The estimated elongation rate is around 4-fold lower in the presence of ribonucleotides (~12 nt/s vs. ~50 nt/s).

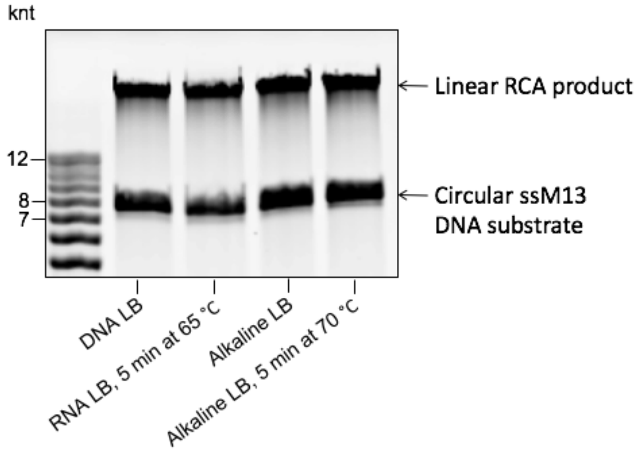

Supplementary Figure 16: Validation of DNA quantitation on agarose gels when using the alkaline treatment. The synthesized ssDNA product of an RCA reaction performed with the purified Φ29 DNAP in a PURE*frex*-like nucleotide pool (dNTPs + NTPs) was subject to different treatments prior loading on gel. No difference is observed, which indicates that under these specific conditions the ssDNA does not undergo cleavage at the site of an incorporated NMP. Therefore, the protocol of alkaline treatment does not bias the amount of synthesized DNA visible on gel. Final loading buffer (LB) conditions are: DNA LB, 5% glycerol; RNA LB, 48% formamide; alkaline LB, 30 mM NaOH. Assessing the misincorporation of NMPs will require the use of stronger alkaline conditions (0.3 M NaOH) combined with a higher temperature during sample treatment [14].

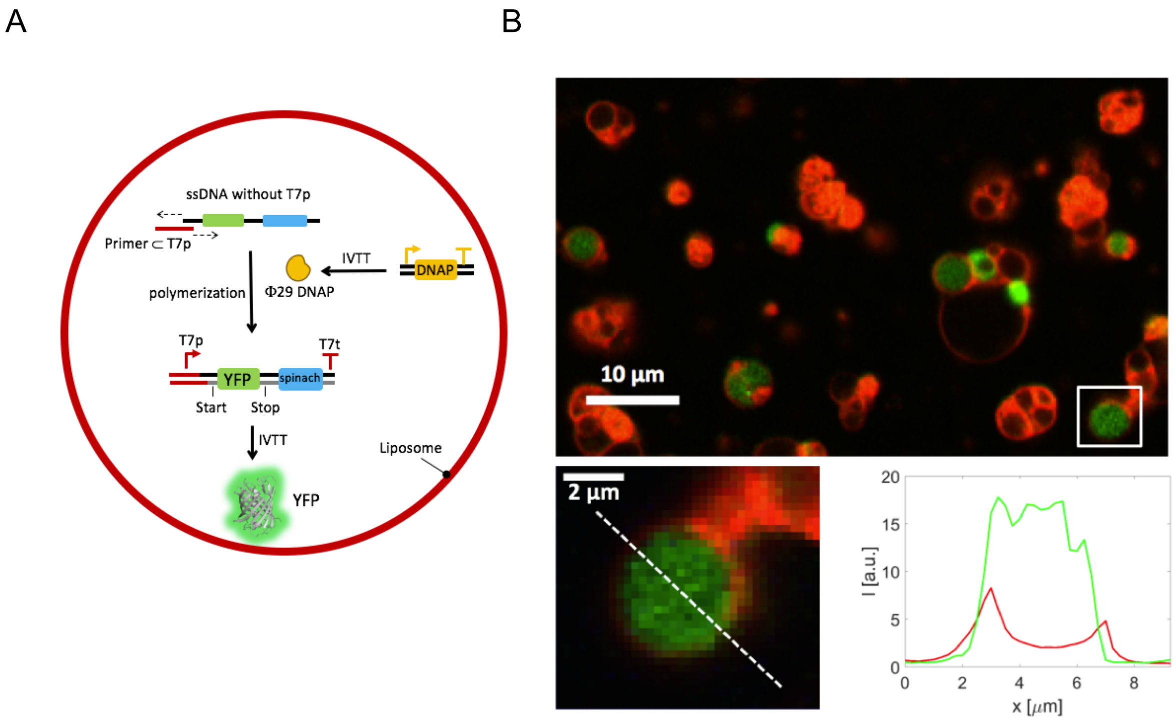

Supplementary Figure 17: The synthesized Φ29 DNAP can elongate a primer-template into transcriptionally active dsDNA inside liposomes. (A) Schematic of the reaction scheme. The overall approach is similar as described in Supplementary Fig. 5 and the protocol is detailed in the Supplementary Methods. (B) Fluorescence micrograph of immobilized liposomes (red membrane dye) with expressed YFP protein displayed in green. A blow-up of one YFP-expressing liposome (framed in the top image) is shown at the bottom, along with the corresponding line intensity profiles of the membrane dye and YFP signals. In a control experiment lacking the Φ29 DNAP gene, only a very few liposomes display YFP fluorescence (not shown), presumably resulting from the expression of carry-over dsDNA from the PCR. Two independent experiments were conducted and a representative field of view is shown. The fluorescence signal from Spinach was weak and is not shown here. a.u., arbitrary units.

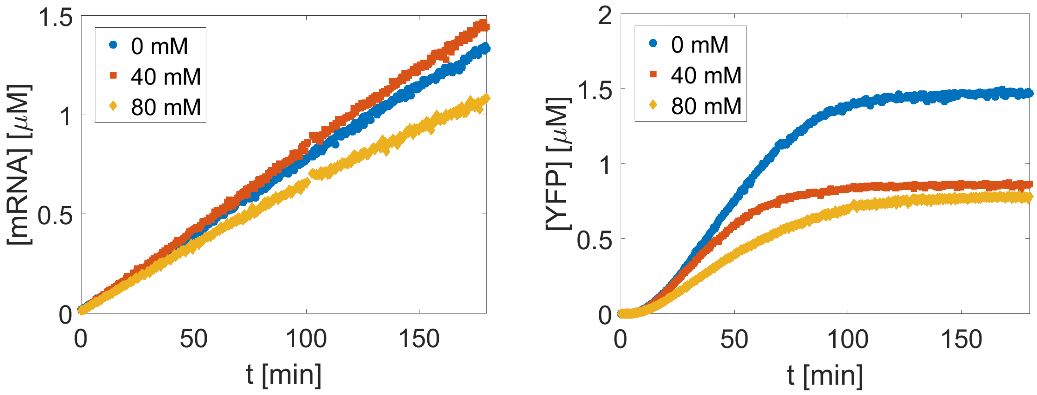

Supplementary Figure 18: Effect of rhamnose on cell-free gene expression. We used the *T7p-eYFPco-LL-Spinach-T7t* dsDNA construct described in Supplementary Fig. 5 (and in its corresponding experimental section) as a template for a gene expression experiment in cuvette. In one sample, 40 mM rhamnose was added, a concentration similar to that expected in the liposome experiments. An even higher concentration was also tested. The kinetics of mRNA and protein production were simultaneously monitored for all three conditions and the time traces are displayed. The results show that rhamnose reduces the yield of synthesized protein by about 40%, whereas transcription is largely unaltered. Despite its negative influence on translation, rhamnose is a necessary solute to assist formation of large unilamellar vesicles. Further investigations to titrate rhamnose concentration are required to see if better compromising conditions can be found.

SUPPLEMENTARY REFERENCES

1. Forster, A. C. & Church, G. M. Towards synthesis of a minimal cell. *Molecular Systems Biology* **2**, 45 (2006).
2. Sakatani, Y., Ichihashi, N., Kazuta, Y. & Yomo, T. A transcription and translation-coupled DNA replication system using rolling-circle replication. *Sci. Rep.* **5,** 10404 (2015).
3. Kita, H., Matsuura, T., Sunami, T., Hosoda, K., Ichihashi, N., Tsukada, K., Urabe, I. & Yomo, T. Replication of genetic information with self-encoded replicase in liposomes. *ChemBioChem* **9,** 2403–2410 (2008).
4. Szostak, J. W., Bartel, D. P. & Luisi, P. L. Synthesizing life. *Nature* **409,** 387–390.
5. Ichihashi, N., Usui, K., Kazuta, Y., Sunami, T., Matsuura, T., Yomo, T. Darwinian evolution in a translation-coupled RNA replication system within a cell-like compartment. *Nat. Commun.* **4,** 2494 (2013).
6. Yumura, M., Yamamoto, N., Yokoyama, K., Mori, H., Yomo, T. & Ichihashi, N. Combinatorial selection for replicable RNA by Qβ replicase while maintaining encoded gene function. *PLoS One* **12,** e0174130 (2017).
7. Attwater, J., Wochner, A. & Holliger, P. In-ice evolution of RNA polymerase ribozyme activity. *Nat. Chem.* **5,** 1011–1008 (2013).
8. Wochner, A., Attwater, J., Coulson, A. & Holliger, P. Ribozyme-catalyzed transcription of an active ribozyme. *Science* **332,** 209–212 (2011).
9. Mencía, M., Gella, P., Camacho, A., de Vega, M. & Salas, M. Terminal protein-primed amplification of heterologous DNA with a minimal replication system based on phage Phi29. *Proc. Natl. Acad. Sci. U. S. A.* **108,** 18655–60 (2011).
10. van Nies, P., Nourian, Z., Kok, M., van Wijk, R., Moeskops, J., Westerlaken, I., Poolman, J. M., Eelkema, R., van Esch, J. H., Kuruma, Y., Ueda, T. & Danelon, C. Unbiased tracking of the progression of mRNA and protein synthesis in bulk and inside lipid vesicles. *ChemBioChem*, **14,** 1963–1966 (2013).
11. van Nies, P., Soler Canton, A., Nourian, Z. & Danelon, C. Monitoring mRNA and protein levels in bulk and in model vesicle-based artificial cells. *Methods in Enzymology* **550**, 187–214 (2015).
12. Paige, J. S., Wu, K. Y. & Jaffrey, S. R. RNA mimics of green fluorescent protein. *Science* **333,** 642–646 (2011).
13. Esteban, J., Blanco, L., Villar, L. & Salas, M. In vitro evolution of terminal protein-containing genomes. *Proc. Natl. Acad. Sci. U. S. A.* **94,** 2921–2926 (1997).
14. Yao, N. Y., Schroeder, J. W., Yurieva, O., Simmons, L. A. & O'Donnell, M. E. Cost of rNTP/dNTP pool imbalance at the replication fork. *Proc. Natl. Acad. Sci. U. S. A.* **110**, 12942–12947 (2013).
